# The Crisis at Camberwell Infirmary

**Published:** 1913-11-08

**Authors:** 


### THE CRISIS AT CAMBERWELL INFIRMARY.

### The Committee's Report on the Medical Staff.

THE Camberwell Board of Guardians, at their meeting on the 29th ult., considered the strained relations between Dr. Keats, the medical superintendent, and his three assistants, referred to in our last issue. The infirmary committee had considered the matter at two meetings, and they presented the following report to the Board :—

"The committee beg to report that they have had under notice a letter addressed to them by Dr. R. G. Riches, assistant medical superintendent, Dr. G. C. Soutter, senior assistant medical officer, and Dr. A. Mearns, second assistant medical officer, stating that they had written Dr. Keats, medical superintendent, asking for an interview with him on the grounds that 'the medical officers, both past and present, have for some considerable time felt that the kindly feeling and proper relations that should exist between the medical superintendent and them as assistant medical officers have been greatly strained,' and that such interview had taken place but no satisfactory conclusion was arrived at, and asking that the committee would take the matter into their consideration.

"The medical superintendent has also brought the letters above referred to under the notice of the committee, and has given them the fullest explanation of the need for the observance of the rules and regulations governing the medical assistants of the infirmary. The committee have interviewed the officers concerned, and beg to report that they have gone carefully into the matters brought to their notice by the several officers, and that as regards Dr. Riches the committee feel that he has failed to appreciate the responsible office of assistant medical superintendent (1) by signing the letter above referred to instead of going alone to the medical superintendent, and more particularly in view of the unsatisfactory replies given by him to certain questions put to him at the committee meeting by the medical superintendent; (2) that he has not set a good example owing to his unpunctuality in performing his duties; (3) that he has lacked the deep thought necessary to one holding his position; (4) that instead of taking a firm attitude in the interests of the institution he has been guided by those not deeply concerned in its welfare. As regards Dr. Soutter: (1) That after being at the infirmary one year and performing excellent work he allows himself to be influenced more particularly by Dr. Mearns, his junior; (2) his unpunctuality in leaving the infirmary in the morning to attend the children's homes; and (3) lack of appreciation of responsibility as shown by him in discussing the present situation with the members of the nursing staff. As regards Dr. Mearns: (1) That he lacks the necessary qualities in the position of anyone holding the post of assistant medical officer; (2) that although new in the service he has been unpunctual on many occasions in commencing his duties; (3) that although the book of rules regarding his work has been given to him, his attention on more than one occasion has had to be called owing to disregard of them; (4) that he has failed to observe a strict silence in the present position of affairs.

"In conclusion of the whole matter, the committee beg to report that in their opinion the assistant medical officers have no just cause of complaint, and that Dr. Keats, in his position as medical superintendent of the infirmary, was fully justified in the action he has taken from time to time respecting the matters brought to the notice of the committee, and that such action was taken solely in the best interests of the institution and patients, and that having carefully weighed the effect of the action of the assistant medical officers and its bearing in all its aspects upon the future discipline of the infirmary, the committee regret that they see no other course than to recommend that Dr. Riches, Dr. Soutter, and Dr. Mearns be asked to terminate their connection with the infirmary medical staff by submitting their resignations."

### Letter from Dr. Riches.

Dr. Riches, writing on behalf of his two colleagues and himself, addressed the following letter to the Board :—

"In view of the fact that there is before the Board a resolution of the infirmary committee requesting us to submit our resignations, we respectively beg to place this letter before you for your consideration. Owing to the publicity given to this matter in the daily and weekly papers, which stated we have been asked to resign on charges of neglect of duties, we feel an infinite amount of harm will be done to our future careers in consequence. We have at no time been informed of any neglect of duties being preferred against us, and we consider that if any such charges have been made, in justice to our future careers, we should have been given every opportunity of answering the same.

"We would respectively point out that in the first place we only asked the medical superintendent to discuss in a friendly way a few of the restrictions placed on our social life. Unfortunately Dr. Keats took a wrong view of the spirit in which we approached him, and in consequence of the attitude adopted by him towards us during the interview, we decided to ask the infirmary committee to consider the matter. We must disclaim any personal animosity against Dr. Keats, having always worked amicably with him, as we now are doing and shall continue to do. At the committee meeting on the 17th inst. we were asked a few questions not dealing in any way with the subject under discussion, and we were asked if we were prepared to send in our resignations. Our reply was in the negative, being astounded at the question, having always believed, and also been individually told by Dr. Keats, our work has been entirely satisfactory. At the infirmary committee meeting on the 22nd inst. we requested a copy of the evidence, of which we knew nothing, submitted against us, but were only told the reasons by which the committee had come to a conclusion. All the statements mentioned were general in character, and contained no specific charge of neglect of duty.

"Dr. Keats has told us individually quite recently that our work was always satisfactorily performed, and he informed me that the infirmary committee, which met on September 10, asked him to convey me their thanks and entire satisfaction for the way in which I had performed the duties of acting medical superintendent. I enclose copies of two testimonials I received as recently as March 29, 1913, one from Dr. Keats and one from the Board, which seem to fully justify the belief that my work and character in general have always been quite satisfactory.

"I write this letter on behalf of Dr. Soutter, Dr. Mearns, and myself, believing, as we do, from the reports in the press, that grave aspersions have been thrown at our characters and also grave charges of neglect of duty alleged against us, of which we have never been informed, that will do our careers, as professional men, incalculable harm. We therefore beg your full consideration of it."

### Dr. Capes' Amendment.

Dr. Capes moved an amendment to the committee's recommendation that all words after "opinion" be omitted, and that the following be substituted : "The kindly feeling and proper relations between the medical superintendent and the assistant medical officers have been strained as the result of the medical officers not carrying out their duties in strict accordance with the rules of the Local Government Board service, and with the agreement under which the medical officers were engaged. That the complaints made by the medical officers as to their social position are of such a trifling nature that they should never have been brought before the infirmary committee, and recommend that Drs. Riches, Soutter, and Mearns be asked to carry out their duties in strict accordance with the rules of the infirmary, and that the medical superintendent be asked to report upon the manner in which these three officers have performed their duties in three months' time."

### "A Man Whom all Respected."

Dr. Capes thought the Board was united in the opinion that the kindly feeling between the medical superintendent and the assistant medical officers had for some time been strained. (Hear, hear.) On the one hand, they had the medical superintendent—a man whom all the Guardians respected—suffering from petty irritation. Rules were broken, and things which ought to have been done had been left undone. On the other side, they had three assistant medical officers suffering from an irritation of quite a different kind. They alleged they were not given sufficient time to play tennis or the piano in the afternoon, and that when they were allowed out three nights weekly they were expected home at half-past twelve in the morning. These were trivial complaints, and they ought never to have been brought forward. They maintained that Dr. Keats had altered the dietary of patients unknown to them, and that he did not speak to them as man to man. He (Dr. Capes) was sure that there was nothing professionally that had been done by the medical superintendent to which exception could be taken by the other doctors. The committee had evidence that rules had been broken and not observed, but they were not of sufficient seriousness for the Board to ask the doctors to resign.

Mr. W. T. Layton seconded the amendment, which was carried by twenty-one votes to five.

Mr. H. H. Edmonds then moved that the matter be referred to a committee of the whole Board, but this was defeated by twenty-three votes to ten.

The resolution of Dr. Capes was then agreed to.

A letter was received from the Local Government Board sanctioning an increase in the salary of Dr. Riches, the assistant medical superintendent, from £190 to £230 a year, rising by £15 a year to £260. When the application for the increase was first considered by the Local Government Board they declined to sanction it; but the Guardians as recently as October 1 pressed the Board to agree to the increase, seeing that Dr. Riches had held this appointment since September 1911.

To the same meeting of the Guardians the Local Government Board forwarded a copy of a petition, signed by 801 ratepayers of Camberwell and others, asking for a public inquiry into the management of the infirmary, particularly with reference to the "flogging child patients," the visitation of children by their parents and friends, and the proposed increase of the salary of the medical superintendent. The Board asked for the observations of the Guardians on the petition, and at the same time drew attention to the regulations relating to punishments in the General Consolidated Order in the institutions.

